# Quantitative Evaluation of Waste Separation Management Policies in the Yangtze River Delta Based on the PMC Index Model

**DOI:** 10.3390/ijerph19073815

**Published:** 2022-03-23

**Authors:** Fang Liu, Zhi Liu

**Affiliations:** School of Economics and Management, Anhui Polytechnic University, Wuhu 241000, China; liufang@ahpu.edu.cn

**Keywords:** waste separation management, PMC index model, quantitative evaluation, optimization path, Yangtze River Delta

## Abstract

Numerous policies have been formulated and implemented to strengthen waste separation management activities in many countries. Waste separation management policies (WSMPs) must be evaluated as the precondition for reducing deviations from policy implementation and improving waste separation performance. Based on text mining technology and the construction of a policy modeling consistency (PMC) index model, we conducted a quantitative evaluation of 22 WSMPs issued by central governmental departments and provinces in the Yangtze River Delta, China from 2013 to 2021 and analyzed their optimization paths. The results suggest that the PMC index of the selected WSMPs has an upward trend. The average PMC index of 22 WSMPs was 6.906, indicating good quality in the policy texts. The PMC index identified seven, nine, five, and one of the policies as being perfect, excellent, good, and acceptable, respectively. The characteristics of WSMPs were further illustrated through PMC surface charts. Based on this, optimization paths for WSMPs with lower PMC indexes are proposed, which indicate that existing WSMPs have great potential for optimization in terms of harsher constraint regulations, context-appropriate incentives, and cultivation of market participants. Finally, this study provides a beneficial reference for similar cities or countries to improve their performance in the management of waste separation and environmental protection.

## 1. Introduction

Policy evaluation refers to producing qualitative and quantitative assessments of the value of formulated policies via certain procedures or techniques to check their effects in practice and determine whether to change or end them [1]. Policy evaluation is an indispensable link in the reasonable allocation of policy resources and scientific formulation of public policies [2], and plays a vital role in governments’ effective social intervention and social public management [3,4].

With the remarkable improvement in the living standards of urban and rural residents, the excessive consumption of various food and electronic products has led to massive production of household waste (HW), which consequently not only poses a huge challenge to public environmental pollution management [5,6] but also has gradually become an important factor constraining sustainable urban development [7]. Many countries in the world are already aware of the environmental and health risk caused by unregulated dumping of waste, and have promulgated many policies to strengthen waste collection, transport, and recycling, which are regarded as one of the most important components of the circular economy [8,9,10]. As the largest developing country, China has also explored effective waste management measures and formulated a series of policies since the last century [11]. These laws and regulations include not only overall objectives, main tasks, and detailed plans for construction of fundamental treatment facilities, but also incentives such as the provision of corresponding subsidies for low-value recyclable recovery, key technology research and development, and waste incineration and power generation [12].

However, these related waste separation management policies (WSMPs) do not fully work in practice due to various reasons: (1) some sanitation enterprises cheat to obtain subsidies by taking advantage of these issued policies, which lead to resource mismatch [13]; (2) residents lack extensive, continuous participation in the whole process of waste management; (3) the rapid rise in the level of urbanization has increasingly integrated rural household waste into urban waste treatment systems, consequently resulting in insufficient processing capacity at the original facilities [14,15]; (4) the boom in shopping online triggers huge demand for containers and brings new challenges in waste management [16]. Good waste management practice depends not only on strong policy executive ability, but also on the quality of the policies formulated by administrative sectors [17,18]. Hence, it is greatly necessary and significant to evaluate the current WSMPs and obtain deep insights in their content to better establish waste separation governance systems, promote the cultivation of waste separation habits, and minimize the negative environmental impact deriving from unregulated waste disposal.

**Figure 1:**
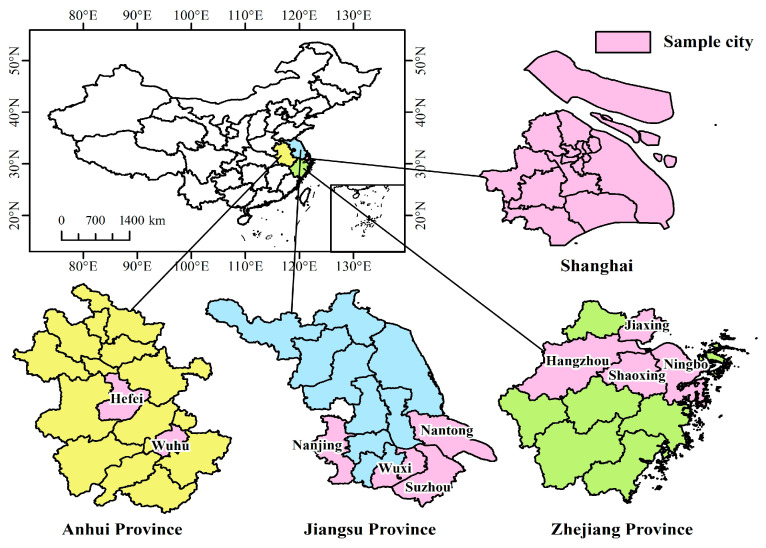
Geographical maps of the Yangtze River Delta region.

Extensive literature has covered the effects of WSMP implementations and their impact on the environment, based on different national backgrounds. However, most researchers conduct qualitative analysis of WSMPs at the macro level. A policy document is an objective, accessible, and traceable written record of the policy system and policy process, which reflects the behavioral imprint of the government’s handling of public affairs. With the advent of the data era, governmental information is increasingly widely disclosed. At the same time, new research methods such as text mining, semantic analysis, and data visualization have been developed [19,20]. All of these provide a broader space for quantitative analysis of public policy. Furthermore, quantitative research on policy helps to open the black box of governments’ decision-making processes, so that public policy is presented to the public not as a “result” but as a “process” [21]. The contributions of this paper are the following: First, it used text mining technology to screen WSMPs in the Yangtze River Delta region (Figure 1) and extract keywords with higher frequency, which helped us quickly understand the main components of each policy. Second, a quantitative evaluation index system was constructed based on the PMC index model, which provides a new research approach for public policy evaluation. Third, the optimization path of each policy was analyzed based on the gap between the second-level variables and the average, which not only provides a theoretical underpinning and standards for revising the current WSMPs, but also promotes increased consistency within a policy cluster.

The remainder of the paper is structured as follows. Section 2 is a literature review. The methodology and data sources are explained in Section 3. In Section 4, the text mining technology is used to categorize the policy texts and extract high-frequency words. Based on this, a PMC index model is constructed to quantitatively evaluate 22 WSMPs before summarizing their characteristics, and then the optimized paths for policies with lower scores are analyzed. Section 5 summarizes the conclusions and policy implications.

## 2. Literature Review

In this section, we review three categories of literature that are most relevant to the present research, namely, the choice of incentive policy or constraint policy for WSMPs, the impacts of WSMP implementation, and evaluations of the WSMPs of different countries.

### 2.1. Selection of WSMPs: Incentive Policy or Constraint Policy

Incentives and constraint regulations are widely formulated by many countries in waste separation policy, involving three basic components: source classification, transfer transportation and terminal treatment. For instance, Japan launched the Container and Packaging Recycling Law and the Household Appliances Recycling Law in 1995 and 1998, respectively, to regulate the disposal of packaging containers, such as PET plastic bottles, and large household appliances. A legal system for the management of waste sorting, with clear responsibilities and strict supervision and punishments, provides external pressure to push citizens to cultivate habits of garbage classification [22]. The harshest punishment for littering residents is five years in prison and a fine of up to 10 million yen [23]. Using simulation scenarios, Ref. [24] found that illegal waste dumping behavior could be vastly restrained by penalties, and the amount of recycled and reused waste could be greatly increased as a result of subsidies. Ref. [25] argued that a balance among government subsidy levels, costs of HW treatment, and profits from waste recycling is important for sustainable HW management. Property service enterprises in Germany are encouraged to provide HW collection containers and specific transport vehicles to ensure that dry waste, wet waste, and hazardous waste can be transferred separately. Ref. [26] suggested that a policy that mixes the charging of a household waste disposal fee with the provision of recycling subsidies is essential for robust waste separation governance. The three ways of waste terminal treatment are incineration, composting and sanitary landfill. The environmental requirements and subsidies for these methods are significantly different across different countries. High pollution from waste incineration exposes communities near incinerators to harmful, costly public health risks. Mercury and dioxins from incinerators can bioaccumulate in fish and other aquatic species, contaminating local and traditional food sources, and potentially causing increases in premature birth rates in women if not adequately treated [27]. Therefore, waste incineration is opposed by many low-income communities in America and alternatives are expected to be presented. Landfill is widely viewed as the least preferable treatment approach, for it may lead to water and soil pollution due to leaching and seepage. The member countries of the EU have been required to reduce the volume of biodegradable waste landfills since the year 1999 [28]. According to the EU’s rules regarding Packaging and Packaging Waste, the packaging design for products must be optimized to prevent the generation of additional packaging waste [29]. Landfill taxation was introduced to price the disposal of HW in the UK, and its elastic characteristics regarding disposal were effective in shifting HW from landfill to incineration [30].

Most of the above-mentioned studies focus on a single incentive-policy or constraint-policy tool and its influence on choices and decision-making, but fewer researchers compare a cluster of policies in terms of design of incentive and constraint instruments. Our study regards this choice as one of the indicators influencing the effectiveness of public policy, and comprehensively evaluates Chinese waste separation policy clusters with the goal of creating a marginal contribution to environmental governance policy optimization.

### 2.2. Impact of WSMP Implementation

The impact of policy implementation tools is reflected in three aspects: changes in the cost of the WSMP, in the administrative management pattern, and in residents’ attitudes or emotional tendencies. For instance, in Japan, the responsibility for HW sorting mainly lies with the residents of each administrative district, which leads to complexity in the front-end classification [31]. The many detailed requirements in the sorting process greatly increase the amount of labor and time demanded of Japanese household members. The costs of front-end collection, supervision, and transportation also increased significantly, further increasing social labor costs [32]. Ref. [33] analyzed the tradeoff between investment in a new waste landfill and policies to increase the recycling rate via construction of a dynamic model, and found that the cost of ignorance regarding waste pollution was higher than that of public policy.

At the same time, comprehensive waste separation governance leads to changes in administrative management patterns, the working efficiency of research and development centers, and the relationships among countries [34,35]. A specific administrative unit or group may be established to implement roles of coordination and supervision in waste management. For instance, local waste generation in the town of Capannori, Tuscany, Italy, was reduced by 40%, and 82% of waste was collected separately, since it signed the EU Zero Waste Strategy Agreement [36]. Bruges, Belgium, established the Bruges Food Lab, with the support of the local municipality’s environmental protection department and civil society organizations, in order to promote the implementation of the “zero food waste” strategy. In many countries, the performance of a waste separation policy is taken into consideration when considering promotions for local officials [37]. In addition, an appropriate mix of policies tends to be one of the important influencing factors of successful waste separation governance. Policy compatibility is worthy of deep consideration when designing multiple waste management policies. Using an experimental methodology, Ref. [38] found that incentive-based HW sorting and recycling policies had the effect of promoting waste reduction and safe treatment.

WSMP exerts great impact on residents’ emotional states. The investigation by [39] showed that most interviewed residents in Shanghai initially felt inconvenienced when they were asked to deposit waste in certain places at certain times, but they felt comfortable and happy after practicing this rule for half a year. Ref. [40] analyzed the comments of users from Sina Microblog using text mining technology, and concluded that nearly half of all citizens hold negative emotions towards local WSMP. The main reasons for resident complaints and irritation were the fines, scheduled waste dumping rules, and improper recycling operations conducted by property companies. Ref. [41] interviewed 330 citizens of Hawassa, Ethiopia, to investigate their willingness to pay for solid waste management, and found that a majority of sampled residents were willing to pay 0.62 USD towards relocation of waste dumping sites and retrofits of old waste vehicles.

A perfect policy does not always translate into a good or effective implementation. Most scholars pay much attention to how policy is carried out in practice and attempts to bridge the gap between the requirements of current policies and the resulting behaviors, but they ignore the quality of the policy itself, which by nature is the precondition for responsible actions. A deep insight into the structure and content of waste separation policies is needed. Our paper attempts to do so taking the Yangtze River Delta as an example.

### 2.3. Evaluation of WSMPs from Different Countries

WSMPs from different countries have been evaluated, and their characteristics discussed, by many scholars. Positive cases such as Japan and the EU and negative cases such as India are summarized in this section in terms of waste management policy. Japan was the earliest country in the world to implement waste separation, and did so most effectively. Its waste treatment policy focus shifted from terminal treatment (before 1970s) to source classification (1970s–1980s), and from waste recycling (1990s) to waste disposal (2000s and later) [42]. The salient features of waste treatment policy in Japan include the establishment of a complete legal system, the fining of polluters, the principle of expanded producer responsibility, strict enforcement of the law, and full social participation [43]. The European Commission proposed a set of standards on waste recycling, requiring its member states to develop their own national waste prevention and control plans on the basis of this standard [44]. The disposal of waste in European countries is affected by the EU’s uniform policy directive. Economic restraint policies such as waste fees and landfill taxes, and economic incentive policies, including concessional loans and subsidies, were widely adopted in the EU. For instance, residents in the Netherlands can obtain subsidies by collecting waste paper, waste metal and other recyclable waste [45]. Citizens in Finland are charged approximately 40% less for separated waste than for mixed waste [46]. By contrast, although many policies were promulgated in India [47], the objectives in the treatment of waste were not achieved due to a lack of appropriate strategies or clarity among stakeholders [48].

Various methods, such as the system dynamics model (SD), the difference-in-differences model (DID), text mining technology, and content analysis, are extensively used in evaluation of waste management policy. For instance, Ref. [49] used a combination of SD and scenario analysis to analyze the expected impact of waste-to-biogas conversion on GHG and PM2.5 emissions based on a case study in Kisumu, Kenya. A “program theory-based” evaluation was conducted by [50] to analyze food waste policy in Italy, and highlighted that donation may be a better option for dealing with surplus food. Ref. [51] argued that there is no one single effective waste management policy that fits all territorial levels after comparison of various predictive models, which indicated that effective efforts for waste sorting were affected by multiple factors at the micro-regional or community level.

The existing policy evaluation literature provides good knowledge for understanding the practical effects of and social influences upon waste management policies across different countries, along with the application of various policy analysis models. However, research focusing on Chinese waste separation policies is still deficient. Hence, to narrow this academic gap, this study focuses on evaluating the consistency of WSMPs in the Yangtze River Delta, China, using the PMC index model to illustrate the advantages and disadvantages of WSMPs and aiming to extend knowledge regarding the basic elements of high-quality policy documents.

## 3. Research Design

### 3.1. The PMC Index Model

Initially proposed by Estrada, the PMC index model has been widely used to evaluate the consistency of public policy, including green development policy [52], plastic bag ban policy [53], and tidal energy development policy [54]. The potential applications of the PMC index model have been extended from exploration to deepening and from single domain to multi-domain. Generally, the overall usage of the PMC index model is now preliminarily standardized, and its advantages are recognized by the academic community. For instance, Refs. [52,55] thought it was a quantitative method that was technically easy to operate. It is widely used in analysis of various industrial development or public management policies, such as safety management policy [56], long-term care insurance policy [57], and pork industry policy [58]. When using the PMC index model, the various influencing factors should first be considered in all directions, to avoid one-sided evaluation to the greatest extent possible. The text mining method can ensure the objectivity and accuracy of the determined variables. This method not only can show the internal consistency level of a policy, but also can reveal the advantages and disadvantages of policy intuitively, providing new ideas and methods for quantitative policy research.

**Figure 2:**
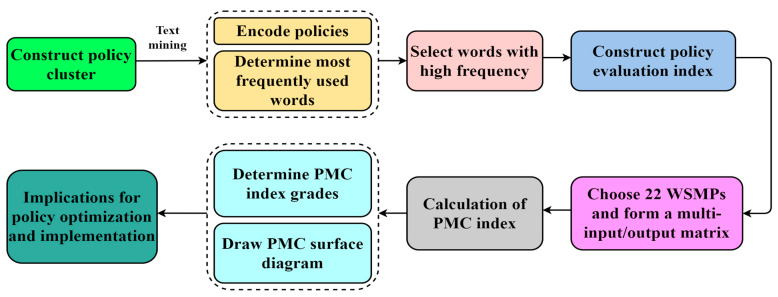
Construction framework for the PMC index model.

Our framework divided the construction of the PMC index model into the following six steps (Figure 2): firstly, the evaluation policy cluster is selected. This model is not a simple quantification of a single policy, but rather a systematic quantitative analysis of many policies [59]. The more policies chosen within a cluster, the more effective the evaluation results could be. Secondly, the evaluation indicators are determined. The evaluation index is a quantitative summary of the selected policy texts. The PMC index model includes N first-level indicators (describing policy level and policy time-effectiveness; for our study, N = 10) and as many second-level indicators as possible, which may then contribute to the achievement of a comprehensive reflection of policy information. Thirdly, text mining technology is used to code policy clauses, and the index parameters are set in binary mode. Fourthly, a multi-input/output matrix is constructed, and the policy hierarchy is sequentially determined. To be specific, the multi-input/output matrix is constructed based on the parameter values of all words extracted from the policy cluster, and good or bad grades on policy are assigned according to the matrix results. Fifthly, representative policy clusters are selected and their PMC index calculated in order to effectively analyze the trend over time associated with the effects of the policy. The last step is to draw the PMC surface diagram of each policy, which helps to describe related issues in WSMP practice. Optimal paths for policy improvement can then be proposed to provide better behavioral guidelines for waste management.

The framework for the PMC index model in the paper is given in Figure 2.

### 3.2. Data Sources for and Construction of the Evaluation Index System

The data in this study were collected from WSMP policy texts obtained from the State Council, the central ministries and commissions, and provinces and cities in the Yangtze River Delta area between 2013 and 2021. The main reason for examining the Yangtze River Delta area as the sample is because it is the most developed area in terms of economic and technological agglomeration in China, with a total population of 227 million and an average urbanization rate of 66.14%. It generates nearly ¼ of China’s GDP and ⅓ of its total import and export volume from less than 4% of its total land area. Due to these characteristics, the amount of household solid waste produced in the Yangtze River Delta area increased from 38.938 million tons in 2015 to 47.635 million tons in 2019, with an average annual growth rate of 5.02%. The Yangtze River Delta area is a well-known national area for establishment and validation of green economic development and ecological management and protection. Therefore, the implementation of WSMPs in the Yangtze River Delta area serves to demonstrate the effects of these policies for other cities or areas. This study on WSMPs in the Yangtze River Delta area is the first step towards exploring successful governance experience in municipal solid waste.

In this study, a total of 190 relevant policies were selected from a search of the PKULAW database and provincial government websites in order to derive policy clusters for quantitative research. These policy texts cover laws, regulations and normative documents dated between 2013 and 2021. Within the policy clusters, 18 documents were normative and departmental working documents from the central government level, while the numbers of local policy documents from Shanghai, Zhejiang, Jiangsu, and Anhui were 37, 51, 48 and 35, respectively.

Text mining technology was used to extract useful information from all policy documents. In detail, ROSTCM software was adopted for this study to encode all policies, segment individual words, and count word frequency [60,61]. After all words were sorted in descending order of frequency, 22 words with high frequency and relevance were selected for further analysis (Table 1).

The 22 selected words can be divided into four groups. The first group of words reflects the goals of waste management, such as ecological preservation, waste reduction, and waste utilization. Within the context of an increasing population and rising consumer demand, the realization of a reduced total volume of waste and an increased use of recycled waste is the basic goal indicator. The final goal is to conserve the ecological environment. The second group of words indicates the content and process of waste management activities, including classification, collection, dumping, disposal, recycling, and transportation. Effective waste separation depends on the completeness of the whole waste management chain and the quality of each element. The criteria for and means of classification, disposal, recycling, and transportation are presented in these policy documents. The third group of words indicates the participants in waste management, such as governments, enterprises, and schools. The government plays the role of leader in solving waste problems, while enterprises act as the main innovative players in recycling waste and developing a circular economy, and students from all educational levels will grow up to be important participants in waste management activities. The Chinese government pays a great deal of attention to strengthening waste classification consciousness among the young generation and cultivating their waste sorting habits via education. The fourth group of words presents specific measures for driving waste management, including propaganda, promotion, encouragement, guidance, and supervision. The frequency of these five words indicates the encouragement of a positive attitude towards waste management in China. Guidance and supervision are indispensable, for most people lack scientific waste sorting knowledge, and their self-discipline may weaken over time.

The PMC evaluation index system for WSMPs was created based on a combination of the classical framework of the PMC index model and the words with highest frequency extracted from the cluster of WSMP documents. The evaluation system was composed of 10 first-level indicators, namely, policy tendency (*X_1_*), policy timeline (*X_2_*), policy responsibility (*X_3_*), policy area (*X_4_*), constraints or incentives (*X_5_*), policy content (*X_6_*), governance scope (*X_7_*), policy tool (*X_8_*), policy type (*X_9_*), and policy accessibility (*X_10_*), which were further subdivided into 32 second-level indicators. The value of each second-level index obeys the distribution of [0,1].

Indicator *X_1_*, policy tendency, was made up of six secondary variables, which can be used to judge whether a policy involves administrative regulation, suggestion, guidance, supervision, promotion planning, or emphasis on implementation. Indicator *X_2_*, policy timeline, was made up of four secondary variables, long-term, medium-term, short-term, or temporary, which indicate the timescale of the policy. All secondary variables of the remaining first-level variables and their associated evaluation criteria are shown in Table 2. The tenth first-level index (*X_10_*) is policy accessibility, which is used to describe the openness of policy to the public. It has no sub-variables.

### 3.3. Calculation of and Effectiveness Criteria for the PMC Index

Besides the above first-level and second-level indexes, two additional parameters were introduced into the structure of the PMC index. If the second-level index could fit into the policy model, this was denoted by “1”; if the second-level index could not fit into the policy model, this was denoted by “0”. That is, each parameter was coded to the binary values “0” or “1”. All second-level indexes had the same level of importance or weight in the multi-input/output table. The first-level indexes were calculated via summarizing all the secondary indexes using Formula (3), and then the final PMC index was obtained by summing up the values of all variables using Formula (4). Finally, the surface graph, to display the resulting PMC index matrix more intuitively, was drawn. The calculations for the PMC surface graph are shown as Formula (5). Based on existing research [55,73,74], the PMC index of WSMPs could be divided into four levels of consistency (Table 3). Specifically, if the PMC index value was less than 3.99, the policy was regarded as having “acceptable consistency”; if the PMC index value was between 4.00 and 6.50, the policy was regarded as having “good consistency”; if the PMC index value was between 6.51 and 7.50, the policy was regarded as having “excellent consistency”; and if the PMC index value was between 7.51 and 8.50, the policy was regarded as having “perfect consistency”. The calculation formulas for the PMC index are detailed below:

*X~N* [0,1], (1)

*X*= {*XR*: [0,1]}, (2)

(3) $$X_{t}(\sum_{j=1}^{n}\frac{X_{tj}}{T_{\left(tj\right)}})\;t=1,2,3,\cdots \infty$$

(4) $$PMC=\left[\begin{array}{c} X_{1}(\sum_{i=1}^{6}\frac{X_{1i}}{6})+X_{2}(\sum_{j=1}^{4}\frac{X_{2j}}{4})+X_{3}(\sum_{k=1}^{5}\frac{X_{3k}}{5})+\\ X_{4}(\sum_{h=1}^{3}\frac{X_{4h}}{3})+X_{5}(\sum_{l=1}^{5}\frac{X_{5l}}{5})+X_{6}(\sum_{m=1}^{11}\frac{X_{6m}}{11})+\\ X_{7}(\sum_{n=1}^{4}\frac{X_{7n}}{4})+X_{8}(\sum_{p=1}^{7}\frac{X_{8p}}{7})+X_{9}(\sum_{q=1}^{6}\frac{X_{9q}}{6})+X_{10} \end{array}\right]$$

(5) $$PMC-surface=\left[\begin{array}{ccc} X_{1} & X_{2} & X_{3}\\ X_{4} & X_{5} & X_{6}\\ X_{7} & X_{8} & X_{9} \end{array}\right]$$

## 4. Results and Discussion

### 4.1. Evaluation Objectives and Empirical Analysis Results

The PMC index model could evaluate the effect of each formulated policy with equal effectiveness. In order to judge the quality of WSMPs and describe their characteristics, a total of 22 representative policies issued in 2013–2021 were selected for the PMC index model (Table 4), denoted P1, P2, P3, P4, P5, P6, P7, P8, P9, P10, P11, P12, P13, P14, P15, P16, P17, P18, P19, P20, P21, and P22 respectively. These 22 policies were issued by the Ministry of Housing and Urban–Rural Development, the National Development and Reform Commission, the local governmental departments of Shanghai, Zhejiang, Jiangsu, and Anhui, and their various subordinate departments. The policies regulate fields such as HW infrastructure construction programs, subsidies for key technology research and development, key HW objectives for public institutions or schools, measures for reducing HW, cultivation of market-based programs for recycling renewable resources, and various other aspects, which together reasonably reflect the effect of WSMPs in the Yangtze River Delta.

Following the evaluation index system and PMC index model, we calculated and confirmed the multi-input/output matrix for the 22 WSMPs, as shown in Table 5. Then, the values of the first-level index for each policy and the corresponding PMC index were calculated. The consistency score for each policy and its corresponding indexes are shown in Table 6.

**Figure 3:**
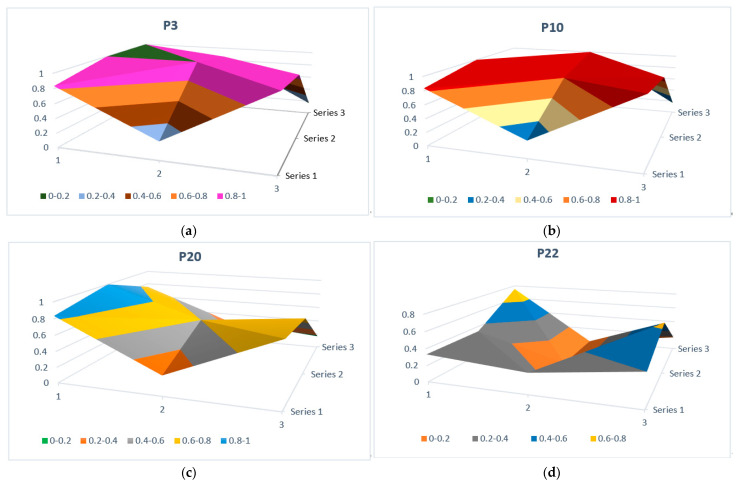
PMC surface charts of (**a**) P3, (**b**) P10, (**c**) P20 and (**d**) P22.

The PMC surface chart for each policy was drawn according to the PMC index matrix. Each PMC surface chart graphically represents the results of the PMC matrix, enabling us to more intuitively see the pros and cons of WSMPs in a graphical context and judge the overall effect of WSMPs. According to [73], if the first-level index value is between 0.9 and 1, then it is of “excellent performance”; if the first-level index value is between 0.7 and 0.89, then it is of “good performance”; if the first-level index value is between 0.5 and 0.69, then it is of “acceptable performance”; if the first-level index value is between 0.3–0.49, then it is of “non-satisfactory performance”; and if the first-level index is between 0–0.29, then it is of “poor performance”. For demonstration purposes, the PMC surface charts for P3, P10, P20 and P22 are displayed (Figure 3). The *x*-coordinates of the matrix are denoted 1, 2, and 3 in the figure, while the *y*-coordinates are denoted series 1, series 2, and series 3. In each graph, a convex part corresponds to a higher PMC index, while a concave part corresponds to a lower PMC index. Evidently, there is not a huge difference between the PMC surface charts for P3 and P10, as they had the same values in second-level indexes *X_1_* (0.833), *X_2_* (0.250), *X_3_* (1), *X_4_* (1), *X_6_* (0.909), *X_8_* (1), and *X_9_* (0.167). P3 and P10 have excellent performance in the policy responsibility, policy area, policy content and policy tool indexes, with values between 0.9 and 1. As seen in the undulation of the PMC surface graph, P20 has fewer convex points than P3 and P10, which indicates that P20 had a lower performance. By contrast, the PMC surface graph of P22 resembles a bowl, with higher values at the margins and lower ones in the middle, because its second-level indexes *X_5_* and *X_8_* were 0, which indicate that P22 had poor performance in the constraints or incentives and policy tool indexes.

**Figure 4:**
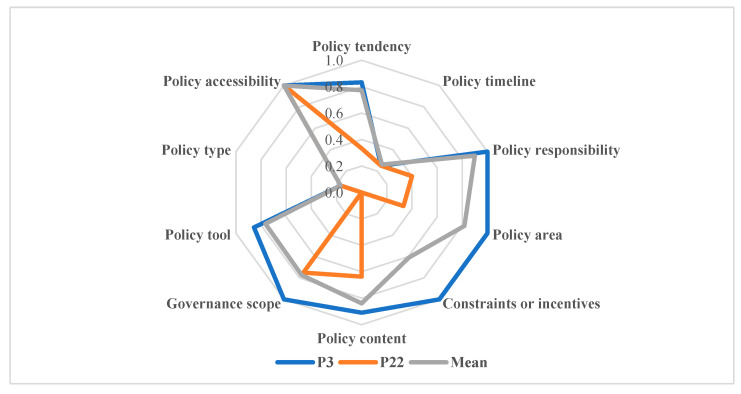
Radar chart comparison of P3 and P22.

In order to perform a clear comparison, the policies with the highest and lowest PMC indexes were further analyzed by backtracking the scores of first-level indexes and second-level indexes. For example, the score differences between P3 and P22 in first-level indexes can be found in Table 6, and are visually shown in Figure 4. On the whole, the PMC indexes of policy responsibility (*X_3_*), policy area (*X_4_*), constraints or incentives (*X_5_*), governance scope (*X_7_*), and policy accessibility (*X_10_*) in policy P3 were all equal to 1. Moreover, the scores of the constraints or incentives (*X_5_*), governance scope (*X_7_*), and policy area (*X_4_*) indexes were much higher than average. By contrast, in the case of P22, the scores of most of its second-level indexes, except for policy type (*X_9_*) and policy accessibility (*X_10_*), were lower than the mean. Great differences existed between P3 and P22 in aspects of the policy responsibility (*X_3_*), policy area (*X_4_*), and policy tool (*X_8_*) indexes. Based on this comparison, we further explored the reasons for differences. In P3, all types of responsible subjects, from government to residents, are included, since this policy is a national guideline for promoting waste sorting management and improving urban service. At the same time, its content reflects a combination of environmental and socioeconomic management. By comparison, P22 is a special notice from a municipal governmental department, which emphasizes that the governmental department must play a leading role in waste management. P3 calls for the use of multiple policy tools including regulation, education, and mechanisms; for example, it proposed to innovate the current working system by introducing professional service companies or adopting types of franchising and leasing. It also emphasized the development of training, professional knowledge, and skills for garbage classification and collection. By comparison, hardly any policy tool is called for in P22.

### 4.2. Quantitative Evaluation Analysis of the 22 WSMPs

#### 4.2.1. Overall Evaluation on WSMPs

By calculating the PMC indexes of the above 22 WSMPs (Table 6) and drawing the corresponding PMC surface charts (e.g., Figure 3), the 22 policies were ranked as P3 > P19 > P12 > P1 > P16 > P18 > P1 > P7 > P10 > P2 > P15 > P21 > P6 > P4 > P5 > P9 > P8 > P20 > P14 > P13 > P17 > P11 > P22.

Based on the policy hierarchy in Table 3, we could divide these policies into four different levels: seven policies were rated at the “perfect” level, including P1, P3, P7, P12, P16, P18, and P19; nine policies were rated at the “excellent” level, including P2, P4, P5, P6, P8, P9, P10, P15, and P21; five policies were rated at the “good” level, including P11, P13, P14, P17, and P20; and only P22 was rated at the “acceptable” level. Among them, P1, P2 and P3 are state-level policies and their PMC indexes were all higher than the average, indicating that the selection and design of indicators for state-level policies abided by the scientific and comprehensive requirements. The state therefore plays a leading role in policies for the prevention and control of household waste pollution.

#### 4.2.2. Specific Evaluation of Each Group of WSMPs

The advantages of the “perfect” policies, the characteristics of the “excellent” and “good” policies, and the optimization paths for WSMPs with lower scores were analyzed as follows.

(1) The “perfect” group of policies

According to the results in Table 6, P3, with a PMC index of 8.016, was the highest-ranked policy. Judging from the content, its core idea is to accelerate the establishment of a waste disposal system that uses separated collection, storage, transportation, and terminal treatment, and to form a waste classification system with the features of a basis in laws and regulations, promotion by the government, and universal resident participation. It requires public institutions (including party and governmental organs, schools, scientific research institutions, cultural, publishing, radio and television institutions, associations, and other community management units) and related enterprises (including hotels, restaurants, shopping centers, supermarkets, professional markets, farmers’ markets, wholesale markets for agricultural products, shops, commercial offices, and so on) to take responsibility for HW disposal. It expects these institutions to develop a supporting system for WSM consisting of a collection and transportation system corresponding to the categorization of waste, a recycling system utilizing renewable resources, terminal treatment facilities linked to waste separation, and cooperative waste disposal and utilization. Evidently, P3 provides the relevant responsible parties with clear objectives, principles, compulsory requirements, and specific measurements for WSM, indicating that this policy was comprehensively and scientifically designed.

P19, announced by the Ningbo municipal government, had a PMC index of 7.916, ranking second. This policy focuses on guiding all public institutions and urban residents to control the increase in HW and to strengthen the recycling of renewable resources. It developed several major tasks consisting of improving the quality of waste classification at the source, constructing a separated collection, transportation, and treatment system, upgrading the existing facilities, improving the system of policies and regulations, promoting waste sorting education, and exploring a joint work model including social workers and volunteers. Most importantly, this policy presents measures for the assessment of urban household waste classification and recycling. The detailed contents involve the subjects of assessment, scope of evaluation, methods of inspection, and scoring standards. The higher score of P19 resulted from the clear assignment of responsibility for each task, and the design of a feasible performance evaluation scheme for the whole WSM process.

P12, ranked third, was announced by the Nanjing municipal government and had a PMC index of 7.850. Moreover, the score of each second-level index was higher than the average score. P12 belongs to regulations formulated by the Nanjing municipal government in accordance with the Law of the People’s Republic of China on the Prevention and Control of Environmental Pollution by Solid Waste. It is a normative document subordinate to this law, used to regulate the market’s waste sorting behavior. Therefore, each citizen in Nanjing city must obey P12; otherwise, the violators would bear certain legal responsibilities. This indicates that “hard” policy tools are indispensable for solving public environmental issues.

The PMC index of P16, ranked fourth, was 7.559. P16 is the implementation plan for the creation of the Nanjing municipal solid waste classification demonstration city. The policy proposes efforts to establish two mechanisms (including a HW points incentive mechanism and a HW reduction mechanism) and three systems (including a waste classification integral management platform, a service system of exchange points, and a system for HW separation in transportation and disposal), and formulate three policies (a recovery treatment subsidy policy for low-value recyclables, subsidies for the local and regional disposal of kitchen waste, and subsidies for the recovery and treatment of electronic and hazardous waste) in order to guide all residents to participate in household garbage classification and environmental pollution reduction. Moreover, it presents several quantitative objectives, involving improving kitchen waste disposal capacity and increasing the coverage rate of the HW sorting points exchange service. The responsible institutions are clearly assigned. Each sector must finish its assigned mission before the deadline.

P18, the Regulations for Municipal Household Waste Classification Management in Ningbo, was reviewed and approved at the 12th meeting of the Standing Committee of the 13th People’s Congress of Zhejiang Province on 31 May 2019. It has the same PMC index value as P16. P18 highlights the establishment of a HW disposal charging system in accordance with the principles “who produces pays” and “produce more, pay more”. It also formulates the methods for implementing the management responsibility area and management responsibility assignation system in detail, listing specific penalty rules.

P1 had a PMC index of 7.558, therefore ranking sixth. Although it was graded “perfect”, its second indexes *X_2_* (policy timeline) and *X_5_* (constraints or incentives) were slightly lower than average. This policy seeks to provide a guideline to encourage relevant interested parties to comprehensively strengthen the scientific management of HW. It suggests efforts to promote waste sorting habits by implementing measures that guide mass participation, introduce waste separation knowledge into school education at all levels, widely mobilize social forces to participate in this activity, and create a good public opinion atmosphere via reporting successful waste classification examples. It also emphasizes the need for accelerated formation of a long-term mechanism to improve charging mechanisms, support capacity through science and technology, and establish a sound mechanism for evaluating the effectiveness of household garbage classification.

P7, ranked seventh, is a notice announced by the Wuhu People’s Government Office. Annual targets for promoting WSM from 2018–2020 are proposed and a HW classification pilot was conducted in different governmental sectors, residential communities, and schools. Most importantly, the municipal finance departments are obliged to provide guaranteed funds for publicizing and teaching the waste sorting requirement. It plans to establish a social supervision mechanism jointly including deputies of people’s congresses, the press, and the public, which will deeply participate in the construction and implementation of the waste sorting system, aiming to form social co-management and joint governance.

(2) The “excellent” group of policies

Among the policies set forth by provinces or cities in the Yangtze River Delta, this section focuses on P5 (Anhui), P9 (Shanghai), P15 (Jiangsu) and P21 (Zhejiang) as examples to elaborate the characteristics or priorities of the policy, and discusses paths towards further improvement.

P5 had a PMC index of 6.983, ranking fourteenth. The scores of second-level indexes *X_4_* (policy area) and *X_7_* (governance scope) were 0.182 and 0.273 lower than the averages respectively. P5 focuses on achieving reductions in waste sources. It encourages people to increase the use of cleaning products and supplies through enforcement of the ban on plastic bags. It calls for hotels, restaurants, and other service industries and enterprises to reduce their supply of disposable goods and promote the use of recyclable goods. Residents are expected to embrace the concepts of low-carbon life and moderate consumption. Postal delivery enterprises and users are guided to use green, recyclable packaging and stuffing in reduced amounts. Although the environmental regulation and social norms are addressed, the development of industrial waste recycling industry is not. It is suggested that P5 expand in terms of policy area and governance scope. For P5, the policy improvement path is *X_7_* → *X_4_*.

P15, issued by Suzhou, had a PMC index of 7.234. The scores of second-level indexes *X_2_* (policy timeline), *X_5_* (constraints or incentives), and *X_6_* (policy content) were 0.023, 0.145 and 0.084 lower than the averages, respectively. P15 designs an action plan for HW separation and disposal in 2019, which falls under short-term policy. Incentive tools such as fiscal subsidies or preferential taxation are absent in this policy. Consequently, it fails to provide institutions, social organizations, and public management units with persistent driving forces. Thus, *X_5_* → *X_6_* → *X_2_* represents the path towards improving P15.

P21, issued by Jiaxing, Zhejiang, has a PMC index of 7.166. The scores of second-level indexes *X_2_* (policy timeline), *X_5_* (constraints or incentives), and *X_7_* (governance scope) were 0.023, 0.145, and 0.023 lower than the averages, respectively. The content of P21 advocates HW source reduction by strengthening the management of construction waste, medical waste, industrial waste, and agricultural production waste, but it fails to provide detailed measures. For example, it intends to establish high standards for a HW sorting demonstration community, but which community would be responsible for this mission is unclear. The regulation is confined to intensifying supervision and inspection. The worst outcomes are subject to criticism or warning, rather than fines. Evidently, neither the restraint mechanism nor the incentive mechanism is satisfactory. It is suggested that P21 follow the improvement path *X_5_* → *X_2_* → *X_7_*.

(3) The “good” group of policies

P11 and P13 had advantages in the policy tool index. P11, guiding all educational institutions to strengthen waste classification, was announced by the Suzhou city government and had a PMC index of 5.668. Seven of the ten first-level variables had lower scores than the average: *X_1_* (policy tendency)—its indicator score was 0.288 lower than average, *X_2_* (policy timeline)—its indicator score was 0.01 lower than average, *X_3_* (policy responsibility)—its indicator score was 0.291 lower than average, *X_4_* (policy area)—its indicator score was 0.182 lower than average, *X_5_* (constraints or incentives)—its indicator score was 0.273 lower than average, X_6_ (policy content)—its indicator score was 0.141 lower than average, *X_7_* (governance scope)—its indicator score was 0.273 lower than average. It is suggested that the initial focus of improvement should be on the policy area, policy timeline, and governance scope indexes. For P11, the policy promotion path is *X_3_* → *X_1_* → *X_5_* → *X_7_* → *X_4_* → *X_6_* → *X_2_*.

Similarly, P13 should be improved in the indexes of *X_3_* (policy responsibility), *X_4_* (policy area), *X_5_* (constraints or incentives), and *X_6_* (policy content). For P14, the improvement path is *X_4_* → *X_7_*, because the scores of its second-level indexes *X_4_* and *X_7_* were 0.455 and 0.273 lower than the averages, respectively. P17 emphasizes the basic requirements for waste classification and designates several tasks including activity organization, responsibility assignation, demonstration of the policy effects by governmental sectors, and policy accessibility and education. However, it fails to provide fiscal subsidies, discount loans, land prioritization, and other relevant supporting items for property enterprises or sanitation companies. It also does not stipulate detailed assessment methods regarding classification efforts. Thus, the improvement path for P17 is *X_4_* → *X_5_* → *X_6_*. P20 is a specific opinion announced by the Zhejiang provincial government, which aims to accelerate the cultivation of market-based solutions for the recycling and utilization of renewable HW resources. The major objective is to establish a whole waste recycling and utilization chain with the characteristics of network-based recycling, convenient service, a sorting facility, automatic separation, and intelligent supervision. However, the policy tendency, policy content and policy tool indexes of P20 are lower than the averages; thus, its policy improvement path is *X_1_* → *X_6_* → *X_8_*.

(4) The “acceptable” policy

P22 had the lowest PMC index value at 3.869 and was graded at the “acceptable” level. The scores of its secondary indexes were the lowest of any of the policy-level indicators. Although it clearly proposes a waste sorting rate, resource utilization rate, and harmless treatment rate, its effectiveness is confined to the last three years. Moreover, the feasibility of the working plan is unclear. It emphasizes the construction of categorized collection, storage, transportation, and disposal systems, but fails to assign this responsibility to any department, nor to provide sufficient subsidies.

### 4.3. Further Discussion

The purpose of promulgating WSMPs is to improve the urban and rural environment, promote resource recycling and utilization, speed up the construction of an ecologically harmonious society, and improve the quality of new urbanization and the level of ecological infrastructure construction. By the first half of 2021, WSMPs in the Yangtze River Delta achieved varying degrees of success. Shanghai’s dry waste control volume was 14,847 t/day, representing a decrease of 28% compared with 2019, while wet waste volume was 10,311 t/day (an increase of 89% compared with 2019) and the volume of recyclable material was 7104 t/day (approximately 1.65 times more than that in 2019) [75]. Zhejiang province built 2698 high-standard classification demonstration communities, 222 sorting centers, and more than 7600 recycling outlets. Moreover, Zhejiang became the first province with a zero HW growth rate in China [76]. In Jiangsu province, a total of 96 domestic waste treatment facilities were established, with a daily treatment capacity of 88,900 t and a total incineration capacity of 71,300 tons per day, ranking first in China [77]. Anhui province put 101 municipal solid waste treatment facilities and 3691 separated waste transportation vehicles into operation, and constructed 42,357 separated waste delivery sites. The designed processing capacity for municipal solid waste in this province reached 57,209 t per day [78]. From these data, it is not difficult to conclude that WSMPs in the Yangtze River Delta region played a great role in the improvement in municipal waste pollution control and the recycling of renewable resources. Among these contributions, policies for reducing waste sources, requirements for the construction of waste sorting facilities, mechanisms for youth volunteer participation, and legal liability regulations were most obvious drivers of improved municipal solid waste governance performance.

However, some problems were also exposed in aspects of policy quality and policy implementation. First, WSMPs in the Yangtze River Delta region vary significantly in terms of comprehensiveness and systematization. Shanghai took the lead in China in the exploration of relevant systems and mechanisms for the disposal of municipal solid waste, and its policy chain from source classification to terminal disposal is basically established. Jiangsu achieved great performance improvements in the construction of waste treatment infrastructure and the innocuous disposal of HW. Zhejiang did an excellent job in controlling waste volume. Anhui was left behind them in terms of quality of WSMPs. Uninformed WSMPs may lead to unchanged levels of waste control capacity and waste management performance.

Second, from the perspective of constraint-based policy tools, some waste separation policies are too general to carry out in practice. General guiding ideas such as creating a role for price adjustment and charging fees according to the type and quantity of waste were stipulated in some policies, but feasible and effective charging standards were not established. Severe punitive measures or restraint mechanisms are absent in most WSMPs.

Third, fiscal revenue and environmental investment place constraints on the implementation of WSM. Economically developed areas can upgrade their infrastructure for waste classification, such as installing smart garbage recycling bins to increase the convenience of waste sorting. However, it is difficult to implement sustainable WSM in economically undeveloped areas without sufficient capital investment. Therefore, to achieve effective governance regarding public environmental problems and to reverse the embarrassing situation of “the active government and the passive citizens” in household garbage classification, policy-makers should review the current policies and make appropriate adjustments.

Similarly, an increase in financial support or tax exemption would be useful for the promotion of WSM. This can motivate companies to engage in waste recycling and utilization for more revenue on the condition of receiving subsidies or tax cuts. R&D subsidies are largely useful for encouraging enterprises to seek technological innovation and to improve the efficiency of resource recovery and utilization. Current incentive tools need to be further improved [79]. According to [79,80,81,82], a points exchange system cannot yield a good incentive effect for all residents, because people with high incomes show no interest in material rewards. In addition, the lack of convenience in exchanging rewards reduces the willingness of residents to sort waste.

Another problem is that the government faces heavy operating cost burdens. Some cities, such as Shanghai and Hangzhou, achieved certain socioeconomic and environmental benefits through waste management in the last two years. For example, “Internet + green account” model adopted by Shanghai is a successful case. A “green account” is designed as an electronic account for garbage classification bonus points. It can record residents’ participation behavior in an Internet-based information management system, encouraging citizens to properly abide by waste management regulations. It attracts residents to take the initiative and positively participate in waste sorting through the path of “classification—points—exchange—benefits”. This method links garbage classification with a positive incentive system to increase the classification rate of HW and promote HW reduction. However, the “green account” model is basically operated by the government. Thus, the continuous operating cost exerts a certain amount of fiscal pressure on the government.

## 5. Conclusions and Implications

### 5.1. Conclusions

In this paper, a combination of the text mining method and the PMC index model were used to evaluate the consistency of WSMPs in the Yangtze River Delta region, China. The characteristics of selected WSMPs were further discussed. The main conclusions are given below.

The policy design in the cases studied was generally reasonable. Among the policy cluster studied, seven policies were rated as “perfect”, nine were rated as “excellent”, five were rated as “good”, and only one was rated as “acceptable”, with the average PMC index being 6.906, indicating that the overall design of the policies was scientific. The advantages of policies with higher PMC indexes lie in good consistency across the secondary indexes, such as multiple policy responsibilities, detailed policy content, appropriate constraints or incentives, reasonable policy timescales, and an exquisite mix of policy tools.

At the same time, there is much room for improvement within current WSMPs in the Yangtze River Delta region. First, harsh regulations or other effective constraint policies were not well designed. Strict constraints could include performance evaluations, institutional charges, demotion of managers, and heavy fines. Among the 22 policies, only five policies included the above constraints. Residents are less likely to abide by the norms for waste classification disposal in the absence of feasible constraints or penalties. Second, it was difficult to find a series of systematic incentive policies. Good incentives can ensure the smooth operation of the whole waste industrial chain, and could include subsidies for purchasing professional equipment or vehicles for waste sorting, collection and transportation, subsidies for critical technology R&D, tax relief, and points exchange mechanisms. Approximately half of the policies examined in this cluster included one or two incentives. The lack of suitable and systematic incentives is an obstacle to the rapid development of the waste sorting and recycling industry. Third, the current WSMPs lack sufficient measures to cultivate multiple market participants in the field of waste classification and recycling, as only two of them offered any market-related content. The fundamental route to effective WSM lies in market reform.

### 5.2. Policy Implications

Based on the above research, the implications for policy optimization are proposed below.

First, feasible constraint policy tools should be more widely explored and strictly implemented in practice. At the legislative level, first, the legal responsibilities of each subject need to be clarified. Moreover, official activities for the promotion of waste classification should be organized to ensure that all participants have an enhanced sense of responsibility for reducing and recycling waste. Secondly, the discretionary standards for punishing illegal behavior should be refined, and authoritative policy interpretation and behavior identification should be developed to help judicial practice. The vitality of the law lies in its implementation. All departments of the government should strengthen their supervision of the implementation of WSMPs, and improve public oversight and feedback channels for WSM. Media and residential oversight can be used to link the effects of implementing waste separation with the evaluations of the performance of governmental officials. Government sectors are supposed to play a guiding, exemplary, and supervisory role in WSM. If administrative officers cannot adequately lead or supervise residents or enterprises, they would be demoted.

Second, based on the perspective of the industrial chain, incentive policy tools should be carefully designed by taking into account the heterogeneity of the various participants. For instance, reasonable basic rules and a convenient point exchange process should be designed to meet the demands of families at all income levels. Multiple additional points could be obtained by residents with high participation in garbage classification and those who make significant WSM contributions; for example, high-value appliances or electrical products could be redeemed for points. A combination of tax and subsidization policies aiming to reduce waste volume could be provided to manufacturers and recyclers, so that expanded production responsibility could be better fulfilled. Enterprises and research institutions should be encouraged to collaborate more extensively in order to develop technological innovations regarding intelligent collection, waste separation, waste recycling, and waste–energy conversion. In addition, spiritual motivation is indispensable, as most people wish to be respected. Awarding certificates or medals to enterprises, communities, and individuals who make great contributions in WSM activities can be widely adopted in practice.

Third, the focus of policy could be shifted away from normative guidance towards market competition. Governmental subsidies or tax incentives are conducive to promoting the implementation of waste classification and the development of renewable resource recycling industries. For instance, Ref. [83] found that a combined optimal solid waste tax and emissions tax allows producers to reduce pollution and to increase waste recycling at the lowest possible cost. Ref. [84] also found that joint subsidy–tax mechanisms can motivate recyclers to extend producer responsibility by adding revenue. However, when subsidies were withdrawn, the performance of the WSM was more likely to decrease. Ref. [85] found that a reasonable subsidy scheme (18.8 EUR/MWh) for a biomethane production plant would increase waste utilization by 75% to reach profitability. This indicates that the related enterprises were not ready for free-market competition. In the future, state-owned enterprises and private capital are actively encouraged to participate in the recycling and utilization of renewable household garbage resources, and diversified market entities are to be vigorously cultivated. Guided by policy support, a backbone group of leading renewable resource recycling enterprises operating at large scales, at high efficiency, and with excellent equipment would be cultivated in order to effectively improve the level of intensification and specialization.

Fourth, knowledge regarding waste classification should be incorporated into the national education system in order to cultivate waste sorting awareness and habits among the young generation. Nowadays, Chinese residents have a certain awareness of waste classification, but it is difficult for most people to practice it continuously. Many residents have little knowledge on waste classification. In order to speed up the formation of this habit, garbage classification should be incorporated into the national education system. In detail, waste classification knowledge should be made a compulsory module in kindergartens, primary and secondary schools. At the same time, many other methods including public service advertisements, knowledge contests, volunteer services, benefit performances, and so on are expected to promote and popularize knowledge regarding waste classification, to gradually increase the awareness of waste separation and the ability of residents in the form of seamless services, and to develop the habit of waste sorting.

## Figures and Tables

**Table 1:** The 22 words with the highest frequency.

Number	Words	Frequency	Number	Words	Frequency
1	Waste classification	7082	12	Promotion	815
2	Disposal	3873	13	Ecological environment	658
3	Classification	3704	14	Government	644
4	Collection	1604	15	Enterprise	542
5	Dumping	1498	16	Reduction	527
6	Management	1464	17	Encouragement	297
7	Recycling	1288	18	Education	297
8	Construction	978	19	Implementation	289
9	Utilization	977	20	School	271
10	Propaganda	874	21	Guidance	191
11	Transportation	840	22	Supervision	174

**Table 2:** Structure of the evaluation index system and criteria for the secondary index.

First-Level Index	Number	Second-Level Index	Number	Evaluation Criteria	References
Policy tendency	*X_1_*	Management	*X_11_*	Determine whether the policy involves administrative regulation, suggestion, guidance, supervision, promotion planning, or emphasis on implementation: if it does, then the value is 1; if not, then the value is 0.	[62]
		Suggestion	*X_12_*		
		Guidance	*X_13_*		
		Supervision	*X_14_*		
		Promotion	*X_15_*		
		Implementation	*X_16_*		
Policy timeline	*X_2_*	Long-term	*X_21_*	Determine whether the policy is carried out over the long term (≥7 years), the medium term (4–6 years), the short term (1–3 years), or a temporary (<1 year) period: if it does, then the value is 1; if not, then the value is 0.	[63]
		Medium-term	*X_22_*		
		Short-term	*X_23_*		
		Temporary	*X_24_*		
Policy responsibility	*X_3_*	Local government	*X_31_*	Determine whether the subject responsible for the policy is local government, enterprises, schools, or urban and rural residents: if it is, then the value is 1; if not, then the value is 0.	[64]
		Enterprises	*X_32_*		
		Schools	*X_33_*		
		Residents	*X_34_*		
Policy area	*X_4_*	Environment	*X_41_*	Determine whether the policy involves the fields of the environment, economy, or society: if it does, then the value is 1; if not, then the value is 0.	[65]
		Economy	*X_42_*		
		Society	*X_43_*		
Constraints or incentives	*X_5_*	Rewards	*X_51_*	Determine whether the policy involves constraints (such as punishments and regulations) or incentives (such as rewards, subsidies, resource or monetary support, or a demonstration effect): if it does, then the value is 1; if not, then the value is 0.	[66]
		Punishment	*X_52_*		
		Subsidy	*X_53_*		
		Support	*X_54_*		
		Demonstration	*X_55_*		
Policy content	*X_6_*	Classification	*X_61_*	Determine whether the content of the policy involves household waste classification rules, recycling activity, transportation, waste reduction, infrastructure construction, criteria for waste classification, waste resource utilization, disposal rules, dumping methods, degradation regulations, or pilot regulations: if it does, then the value is 1; if not, then the value is 0.	[67,68,69,70]
		Recycling	*X_62_*		
		Transportation	*X_63_*		
		Reduction	*X_64_*		
		Construction	*X_65_*		
		Criteria	*X_66_*		
		Utilization	*X_67_*		
		Disposal	*X_68_*		
		Dumping	*X_69_*		
		Degradation	*X_610_*		
		Pilot	*X_611_*		
Governance scope	*X_7_*	Country	*X_71_*	Determine whether the policy is enacted at the level of the nation, a province or city, or a county or town: if it is, then the value is 1; if not, then the value is 0.	[70]
		Province	*X_72_*		
		City	*X_73_*		
		County/Town	*X_74_*		
Policy tool	*X_8_*	Responsibility	*X_81_*	Determine whether the policy tool involves responsibility, regulation, propaganda, education, evaluation, or a mechanism: if it does, then the value is 1; if not, then the value is 0.	[69]
		Regulation	*X_82_*		
		Propaganda	*X_83_*		
		Education	*X_84_*		
		Evaluation	*X_85_*		
		Mechanism	*X_86_*		
Policy type	*X_9_*	Opinion	*X_91_*	Determine whether the type of policy is categorized as opinion, announcement, official reply, ordinances, method, or plan: if it is, then the value is 1; if not, then the value is 0.	[71]
		Announcement	*X_92_*		
		Official reply	*X_93_*		
		Ordinances	*X_94_*		
		Method	*X_95_*		
		Plan	*X_96_*		
Policy accessibility	*X_10_*	/	/	Determine whether the policy is open to the public; if it is, then the value is 1; if not, then the value is 0.	[72]

**Table 3:** Consistency categories for WSMPs.

**PMC Index**	0–3.99	4.00–6.50	6.51–7.50	7.51–8.50
**Policy Consistency**	Acceptable	Good	Excellent	Perfect

**Table 4:** The selected WSMPs in this study.

Code	Policy Name	Document Number	Date Issued
P1	Notice of the Ministry of Housing and Urban–Rural Development, the Central Publicity Department, and the Central Civilization Office issuing several opinions on further promoting the work of household waste classification	No.93 [2020] of Ministry of Housing and Urban–Rural Development	27 November 2020
P2	Notice of the National Development and Reform Commission and the Ministry of Housing and Urban–Rural Development on printing and distributing the Development Plan for Urban Household Garbage Classification and Treatment Facilities in the 14th Five-Year Plan period	No.642 [2021] of the National Development and Reform Commission	6 May 2021
P3	Notice of the General Office of the State Council on forwarding the Implementation Plan of the Household Garbage Classification System of the Ministry of Housing and Urban–Rural Development and the National Development and Reform Commission	No.26 [2017] of the General Office of the State Council	18 March 2017
P4	Notice of the General Office of Anhui Provincial People’s Government on further strengthening the Work of household Garbage classification	No.176 [2017] of General Office of Anhui Provincial People’s Government	6 July 2017
P5	Agreement among various government and nongovernmental organizations of Anhui Province, including the Department of Housing and Construction, Development and Reform Commission, Ecological Environment Agency, Business Hall, Department of Education, Administration Organization, Communist Youth League, and Women’s Federation to print and distribute the Anhui province implementation plan for promoting urban household waste classification	No.108 [2019] of Anhui Provincial Department of Housing and Urban–Rural Development	20 March 2018
P6	Regulations of Hefei Municipal Household Garbage Classification Administration	/	1 December 2020
P7	Notice of the Wuhu Municipal People’s Government Office on printing and distributing the Implementation Plan for Household Garbage Classification in Wuhu	No.291 [2020] of Wuhu Municipal People’s Government Office	18 December 2018
P8	Notice of the Shanghai Municipal Household Garbage Classification Reduction Promotion Joint Conference Office on printing and distributing the Effective Comprehensive Evaluation Measures for Household Garbage Classification in 2021	No.2 [2021] of Shanghai Municipal Household Garbage Classification Reduction Promotion Joint Conference Office	9 February 2021
P9	Measures of Shanghai Municipality for promoting reductions in and classification of household garbage	No.14 [2014] Order of the Shanghai Municipal People’s Government	22 February 2014
P10	Notice of the Huangpu District People’s Government Office on printing and distributing the Three-Year Action Plan (2018–2020) for Garbage Classification, Reduction and Comprehensive Treatment in Huangpu District	No.32 [2018] of Huangpu District People’s Government Office	30 June 2018
P11	Notice of the Suzhou Education Bureau and Suzhou City Planning Municipal Administration on printing and distributing the Implementation Plan for School and Household Garbage Classification in Suzhou	No.27 [2018] of Suzhou Education Bureau	29 September 2018
P12	Measures of the Nanjing Municipal Household Garbage Classification Administration	No.292 [2020] of Order of Nanjing Municipal People’s Government	1 June 2013
P13	Notice of the CPC Wuxi Municipal Committee and Office of the Wuxi Municipal People’s Government on the Implementation Plan for the Activity “Garbage Sorting—Public Institutions Taking the Lead in Action”	No.52 [2017] of Wuxi Municipal Party Committee Office	16 August 2017
P14	Notice of the Nantong Municipal Government Office on printing and distributing the Pilot Program for Urban Household Garbage Classification Management in Nantong City	No.171 [2015] of Order of Taizhou People’s Government	6 November 2015
P15	Notice of the Suzhou Municipal People’s Government Office on printing and distributing the Work Action Plan for Household Garbage Classification and Disposal in Suzhou in 2019	No.27 [2019] of Suzhou Municipal People’s Government Office	13 February 2019
P16	Notice of the Nanjing Municipal Government on printing and distributing the Implementation Plan for the Municipal Creation of the Nanjing National Household Waste Classification Demonstration City	No.253 [2015] of Nanjing Municipal Government	14 December 2015
P17	Notice of the Government Offices Administration of Jiangsu Province, Housing and Urban–Rural Development Department of Jiangsu Province, Development and Reform Commission of Jiangsu Province, Publicity Department of CPC Jiangsu Provincial Committee, Education Department of Jiangsu Province, Commerce Department of Jiangsu Province, and Health and Family Planning Commission of Jiangsu Province on promoting the Domestic Garbage Classification project of the Party and Government Organs and other public institutions in Jiangsu Province	No.93 [2017] of Jiangsu Provincial Government Offices Administration	27 December 2017
P18	Regulations of the Ningbo Municipal Household Garbage Classification Administration	No.15 [2019] of Announcement of the Standing Committee of Ningbo Municipal People’s Congress	31 May 2019
P19	Notice of the General Office of Ningbo Municipal People’s Government on printing and distributing the Ningbo Implementation Plan for Municipal Household Garbage Classification, Treatment and Recycling in 2016	No.46 [2016] of General Office of Ningbo Municipal People’s Government	22 March 2016
P20	Opinions of the leading Group Office of Household Garbage Classification of Zhejiang Province, the Department of Commerce of Zhejiang Province, and the Development and Reform Commission of Zhejiang Province on accelerating the cultivation of market-based programs for the recycling and utilization of renewable household garbage resources	No.59 [2019] of Shaoxing People’s Government Office	5 November 2019
P21	Notice of Jiaxing Municipal People’s Government on printing and distributing the Implementation Ideas for Promoting the Classification of Urban and Rural Household Garbage in Jiaxing	No.28 [2017] of Jiaxing Municipal People’s Government	22 August 2017
P22	Notice of the Shaoxing Municipal People’s Government Office on printing and distributing the Shaoxing Urban Household Garbage Classification and Treatment Three-Year Action Plan (2020–2022)	No.36 [2019] of Shaoxing People’s Government Office	16 December 2019

**Table 5:** The multi-input/output matrix of the 22 WSMPs.

Index		P1	P2	P3	P4	P5	P6	P7	P8	P9	P10	P11	P12	P13	P14	P15	P16	P17	P18	P19	P20	P21	P22
*X*_*1*_	*X*_*11*_	1	1	1	1	1	1	1	1	1	1	1	1	1	1	1	0	1	1	1	1	1	1
	*X*_*12*_	0	0	0	0	0	0	0	0	0	0	0	0	0	0	0	0	0	0	0	0	0	0
	*X*_*13*_	1	1	1	1	1	1	1	0	1	1	0	1	1	1	1	1	1	1	1	1	1	0
	*X*_*14*_	1	1	1	1	1	1	1	1	1	1	0	1	1	1	1	1	1	1	1	1	1	0
	*X*_*15*_	1	1	1	1	1	1	1	1	1	1	1	1	1	1	1	1	1	1	1	1	1	1
	*X*_*16*_	1	1	1	1	1	1	1	1	1	1	1	1	1	1	1	1	1	0	1	1	1	0
*X*_*2*_	*X*_*21*_	1	1	0	0	1	1	1	1	0	0	0	1	0	0		1	1	1	1	1	0	0
	*X*_*22*_	0	0	1	1	0	0	0	0	1	1	1	0	1	1	0	0	0	0	0	0	1	1
	*X*_*23*_	0	0	0	0	0	0	0	0	0	0	0	0	0	0	1	0	0	0	0	0	0	0
	*X*_*24*_	0	0	0	0	0	0	0	0	0	0	0	0	0	0	0	0	0	1	0	0	0	0
*X*_*3*_	*X*_*31*_	1	1	1	1	1	1	1	1	1	1	1	1	1	1	1	1	1	1	1	1	1	1
	*X*_*32*_	1	0	1	1	1	0	1	1	0	1	0	1	1	1	1	1	1	1	1	1	1	0
	*X*_*33*_	1	1	1	1	1	1	1	1	1	1	1	1	1	1	1	1	1	1	1	1	1	1
	*X*_*34*_	1	0	1	1	1	1	1	1	1	1	1	1	1	1	1	1	1	1	1	0	1	0
*X*_*4*_	*X*_*41*_	1	1	1	1	1	1	1	1	1	1	1	1	1	0	1	1	1	1	1	1	1	1
	*X*_*42*_	1	1	1	1	0	0	1	0	0	1	0	1	0	0	1	1	0	1	1	1	1	0
	*X*_*43*_	1	1	1	1	1	1	1	1	1	1	1	1	1	1	1	1	0	1	1	1	1	0
*X*_*5*_	*X*_*51*_	0	1	1	1	1	1	1	0	1	1	1	1	1	1	0	1	1	1	1	1	1	0
	*X*_*52*_	0	0	1	0	1	1	0	1	0	1	0	1	0	0	1	0	0	1	1	0	0	0
	*X*_*53*_	0	1	1	0	0	0	1	1	0	1	0	0	0	1	0	1	0	0	1	1	0	0
	*X*_*54*_	1	1	1	1	1	1	0	0	1	0	0	1	0	0	0	1	0	1	1	0	0	0
	*X*_*55*_	1	1	1	1	1	1	1	1	1	1	1	0	1	1	1	1	1	1	1	1	1	0
*X*_*6*_	*X*_*61*_	1	1	1	1	1	1	1	1	1	1	1	1	1	1	1	1	1	1	1	1	1	1
	*X*_*62*_	1	1	1	1	1	1	1	1	1	1	1	1	1	1	1	1	1	1	1	1	1	1
	*X*_*63*_	1	1	1	1	1	1	1	1	1	1	1	1	1	1	1	1	0	1	1	1	1	1
	*X*_*64*_	1	1	1	1	1	1	1	1	1	1	1	1	1	1	0	1	1	1	1	0	1	0
	*X*_*65*_	1	1	1	1	1	1	1	1	1	1	1	1	1	1	1	1	1	1	1	1	1	1
	*X*_*66*_	1	1	1	1	1	1	1	1	1	1	0	1	0	1	1	1	1	1	1	1	1	0
	*X*_*67*_	1	1	1	1	1	1	1	1	1	1	1	1	1	1	1	1	1	1	1	1	1	1
	*X*_*68*_	1	1	1	1	1	1	1	1	1	1	1	1	1	1	1	1	1	1	1	1	1	1
	*X*_*69*_	1	1	1	1	1	1	1	1	1	1	1	1	1	1	1	1	1	1	1	0	1	1
	*X*_*610*_	0	0	0	0	1	0	0	0	0	0	0	1	0	0	0	0	0	0	0	0	0	0
	*X*_*611*_	1	1	1	1	0	0	1	0	0	1	0	1	0	1	0	0	0	0	1	1	1	0
*X*_*7*_	*X*_*71*_	1	1	1	1	1	1	1	1	0	0	1	1	1	1	1	1	1	1	1	1	1	1
	*X*_*72*_	1	1	1	1	0	1	1	1	1	1	1	1	0	0	1	1	1	1	1	1	1	1
	*X*_*73*_	1	1	1	1	1	1	1	1	1	1	0	1	0	1	1	1	0	1	1	1	1	1
	*X*_*74*_	1	1	1	1	0	0	0	0	1	0	0	1	1	0	1	1	0	0	1	0	0	0
*X*_*8*_	*X*_*81*_	1	1	0	0	0	1	1	0	0	1	1	1	0	0	1	1	1	1	1	0	1	0
	*X*_*82*_	1	0	1	0	1	1	1	1	1	1	1	1	1	1	1	1	1	1	1	0	1	0
	*X*_*83*_	1	1	1	0	1	1	1	1	1	1	1	1	1	1	1	1	1	1	1	0	1	0
	*X*_*84*_	1	1	1	0	1	1	1	0	1	1	1	1	1	1	1	1	1	1	1	0	1	0
	*X*_*85*_	1	0	1	1	1	1	1	1	1	1	1	1	1	1	0	0	1	1	1	1	1	0
	*X*_*86*_	1	1	1	1	1	1	1	1	1	1	0	1	0	0	1	1	0	0	0	0	0	0
*X*_*9*_	*X*_*91*_	0	0	0	0	0	0	0	0	0	0	0	0	0	0	0	0	0	0	0	1	0	0
	*X*_*92*_	1	1	1	1	1	0	1	1	0	1	1	0	1	1	1	1	1	0	1	0	1	1
	*X*_*93*_	0	0	0	0	0	0	0	0	0	0	0	0	0	0	0	0	0	0	0	0	0	0
	*X*_*94*_	0	0	0	0	0	0	0	0	0	0	0	0	0	0	0	0	0	0	0	0	0	0
	*X*_*95*_	0	0	0	0	0	1	0	0	0	0	0	0	0	0	0	0	0	1	0	0	0	0
	*X*_*96*_	0	0	0	0	0	0	0	0	1	0	0	1	0	0	0	0	0	0	0	0	0	0
*X*_*10*_	*X*_*101*_	1	1	1	1	1	1	1	1	1	1	1	1	1	1	1	1	1	1	1	1	1	1

**Table 6:** PMC indexes and evaluation criteria of all WSMPs.

Index	P1	P2	P3	P4	P5	P6	P7	P8	P9	P10	P11	P12
*X_1_*	0.833	0.833	0.833	0.833	0.833	0.833	0.833	0.667	0.833	0.833	0.500	0.833
*X_2_*	0.250	0.250	0.250	0.250	0.250	0.250	0.250	0.250	0.250	0.250	0.250	0.250
*X_3_*	1	0.600	1	1	1	0.800	1	1	0.800	1	0.600	1
*X_4_*	1	1	1	1	0.667	0.667	1	0.667	0.667	1	0.667	1
*X_5_*	0.400	0.800	1	0.600	0.800	0.800	0.600	0.600	0.600	0.800	0.400	0.600
*X_6_*	0.909	0.909	0.909	0.909	0.909	0.818	0.909	0.818	0.818	0.909	0.727	1
*X_7_*	1	1	1	1	0.500	0.750	0.750	0.750	0.750	0.500	0.500	1
*X_8_*	1	0.714	0.857	0.286	0.857	1	1	0.714	0.857	1	0.857	1
*X_9_*	0.167	0.167	0.167	0.167	0.167	0.167	0.167	0.167	0.167	0.167	0.167	0.167
*X_10_*	1	1	1	1	1	1	1	1	1	1	1	1
PMC index	7.558	7.273	8.016	7.045	6.983	7.085	7.509	6.633	6.742	7.459	5.668	7.850
Policy grade	P	E	P	E	E	E	P	E	E	E	G	P
Rank	6	9	1	13	14	12	7	16	15	8	21	3
Index	P13	P14	P15	P16	P17	P18	P19	P20	P21	P22	Mean	
*X_1_*	0.833	0.833	0.833	0.667	0.833	0.667	0.833	0.833	0.833	0.333	0.773	
*X_2_*	0.250	0.250	0.250	0.250	0.250	0.50	0.250	0.250	0.250	0.250	0.261	
*X_3_*	0.800	1	1	1	1	1	0.900	0.800	1	0.40	0.900	
*X_4_*	0.667	0.333	1	1	0.333	1	1	1	1	0.333	0.818	
*X_5_*	0.400	0.600	0.400	0.800	0.400	0.800	1	0.600	0.400	0	0.609	
*X_6_*	0.727	0.909	0.727	0.818	0.727	0.818	0.909	0.727	0.909	0.636	0.839	
*X_7_*	0.500	0.500	1	1	0.500	0.750	1	0.750	0.750	0.750	0.773	
*X_8_*	0.714	0.714	0.857	0.857	0.714	0.857	0.857	0.285	0.857	0	0.766	
*X_9_*	0.167	0.167	0.167	0.167	0.167	0.167	0.167	0.167	0.167	0.167	0.167	
*X_10_*	1	1	1	1	1	1	1	1	1	1	1	
PMC index	6.058	6.306	7.234	7.558	5.924	7.557	7.916	6.412	7.166	3.869	6.906	
Policy grade	G	G	E	P	G	P	P	G	E	A	/	
Rank	19	18	10	4	20	4	2	17	11	22	/	

Note: Perfect = P, Excellent = E, Good = G, Acceptable = A.

## Data Availability

The datasets used or analyzed during the current study are available from the corresponding author on reasonable request.

## Nomenclature

WSMPs	Waste separation management policies
WSM	Waste separation management
PMC	Policy modeling consistency
HW	Household waste
CG	Central government
PPW	Packaging and Packaging waste
SD	System dynamics model
DID	Difference-in-differences model
